# Bibliography

**Published:** 1887-08

**Authors:** 


### BIBLIOGRAPHY.

JOSEPH JONES ON FEVERS. MEDICAL AND SURGICAL MEMOIRS: VOL. II, 1887—Containing investigations on the Geographical Distribution, Causes, Nature, Relation and Treatment of Various Diseases, 1855 to 1886. By Joseph Jones, M. D., Prof. Chemistry and Clin., Medicine, Medical Department University of La. Visiting Physician to Charity Hospital; Honorary Fellow Med. Soc. of Virginia. Formerly Surgeon P. A. C. S. Ex-Prest. La. State Bd. Health, (1880-1884) Associate Fellow of the College of Philadelphia; Member of the American Med. Assn; Vice-Prest. Med Society La., [now Prest.] etc.

Vol. II Contains Researches on the origin and effects of Endemic Epidemic, Infectious and Contagious Diseases, Investigations on the Nature and Causes, Relations and Treatment of Malarial (paroxysmal) fever; Intermittent, Remittent, Pernicious, and Hæmorrhagic Malarial Fevers. Comparative Pathalogical Anatomy, Malarial, Typhoid, and Yellow Fevers: Indigenous Remedies of the Southern States; Albinism in the negro race, Oriental Leprosy; Elephantiasis Græcorum: Elephants Leg. (Elephantasis Arabum) New Orleans, La. Joseph Jones, M. D., 156 Washington Avenue, Cor. Camp St., 4th District, 1887, page 1332.

It will be seen that Dr. Jones undertook an almost inexhaustible subject, each separate branch or sub-heading of which would furnish fruitful theme for a large book; and although this volume contains but 1338 pages, besides 16 pages of index, the painstaking author has not slighted any essential detail of any one or part of one subject, but has treated all in a thorough and exhaustive manner. The book is a mine of literary treasures, an "encyclopedia of useful information," and will furnish reading of a character and variety that will not easily sate the earnest student of the important subjects therein discussed. This volume is not, in any sense, a compilation, or even a review of antecedent literature, but is the outcome of years of earnest and patient observation, investigation and experiment, in a field which, to other than an earnest seeker after truth, offers few attractions. Indeed, it may be safely assumed that there is no fun in making post mortem examinations on yellow fever cadavers in a dead house 100 years old, and in an atmosphere reeking with infection and under a temperature of 100 in the shade. Yet a large share of this valuable information could be obtained in no other way; and the interest of science and the demands of humanity required that it should be learned.

Dr. Jones, had he contributed naught else to the medical literature of the country and the day, deserves lasting gratitude for his researches on the medicinal properties of the indigenous plants of the Southern States. These were made during the war when the Confederate army, and the people were thrown on such resources for drugs and medicines; and an undreamed of wealth was thus and then discovered in our fields and forests.

There is much that is curious, as well as interesting in this monument of patience and painstaking labor, and the profession is largely indebted to Prof. Jones for many of the most important revelations in the pathology of the Southern fevers. To do full justice to such a work would require more time and space than we have at our disposal, and we present the book to our readers, as one of rare merit and well worthy of a careful perusal. Like many other enterprises by which the public are benefitted, this proved to be disastrous, financially, to the talented author.

---

A TEXT-BOOK ON SURGERY, General, Operative and Mechanical. By Jno. A. Wyeth, M. D., Prof. of Surgery in the New York Polyclinic, Surgeon to Mt. Sinai Hospital, etc., etc. New York: D. Appleton & Co., 1, 3 & 5 Bond St., 1887. Profusely illustrated with wood cuts and colored plates. Royal octavo, pages–765, Sheep, $8.00. Complete in one volume.

Book reviews by the average medical journal have come to mean just nothing at all, of late years,—especially since the practice has obtained of supplying all publications to all journals,—free of cost, and even of carriage.

It seems that many of our colleagues feel a sense of obligation, which must be requited by a complimentary notice. Everything is complimented. Every book that is issued "fills a long-felt want"; and "no physicians library is complete without it." Those expressions have become as stereotyped as the "busy practitioner"; and having answered this purpose several decades, should now be discarded, and some other mode of expression adopted. We mean to say, there is no, or not sufficient, discrimination, in recommending books—by the medical press. We have been surprised, not to say disgusted,at the fulsome praise bestowed upon books which, in our judgement, had nothing to recommend them, except the binding. But, "comparisons are odious."

The work before us is, indeed, one of rare merit. It is literally, a departure from the beaten track of ages,—a pleasing variation,—an utter repudiation of the stereotyped plan upon which Text Books on Surgery have been built for the last fifty years.—Erichsen, Gross, Druitt, Velpeau and others published admirable "Systems of Surgery," beginning with Histology, and laborious and not very lucid definitions of Inflammation. The student, (they were all written for first course students, and no knowledge whatever, of the subject, was presupposed, on the part of the reader) was carried through "gunshot wounds," "incised wounds," "lacerated and centused wounds," "fractures," "dislocations," "hæmorrhage", etc., etc., like a pupil at dancing school, through the figures of a quadrille,— always in the same relative order,—never any variation under any circumstances. The next man who wrote a "System of Surgery" and had anything new to add,—(and there is and was always something to add) simply *added it* to what had gone before; and the next and the next,— till a "System of Surgery" came to mean a dozen immense volumes,—that no one could attempt to read through.

It is strange, in these days of revolution in pathology, and in nearly everything else,—of progress, of independent investigation, of multiplied facilities, how authors will cling to old ideas, and to the stereotyped old plan of constructing a book ! — "Our thoughts still cling to the mouldering past," — indeed they do—in book-making !

Prof. Wyeth seems to have thrown away all antecedent work, and to have struck out for himself, on a new route. (By the bye, we believe we *do* recognize, in the cuts of " Talipes," and of hip dislocation, some familiar old friends of the days of Druitt.) His book seems to have been written from the standpoint of personal observation, and experience. He starts out just as if no other work on surgery had gone before; and he begins (where most other text books usually wind up) with the *preparation* for an operation, and the *etceteras*; begins by telling the reader what it is necessary to have, or to prepare, who expects to practice surgery; and—since the advent of the germ theory—these appliances have multiplied till they are bewildering. Then he discusses anæsthetics, and tells all about them; and about the accidents which are liable to occur, under their use; and how to meet those accidents. It would be an endless undertaking to touch on every detail of the work, let alone describing it, so we will not attempt it.

This model work brings the student up to the present moment, giving all that is new, and discarding most that is old, or even questionable. It embodies all the latest phases of treatment, as now practiced in the great *modern* hospitals of the great medical center of America. We believe the work will be hailed with delight in Europe, as a splendid exponent of American surgery of to-day.

So great has been the progress in operative surgery, and so many new things have been added lately, that it is a positive luxury to read this book; and to the enthusiast in surgery, or to him who has a just appreciation of its principles, and a pride in his knowledge, it will be a treasure.

Everything is illustrated, from the scalpel and how to hold it, to the most complicated apparatus; from an incision, to the finished operation with the bandage applied; even to the practical application of Esmarch's bandage (the principle)—and the arteries and veins are colored, in all the plates, which gives not only a better idea of the relation of parts, but a pleasing appearance, a finish to the book, mechanically. Then, again (it *should* have been called a "manual," and *not* a text book,—tho' one of our New York contemporaries says it is "an elaborate treatise on surgery"), it is not so bulky as to frighten one out of the inclination to tackle it; nor so unwieldly as to fatigue one to handle it, as are some we wot of —another advantage.

The profession owe much to Prof. Wyeth for having thus made his wonderful experience and observation,—almost his phenomenal skill—available to the most remote doctor. He has left a legacy to posterity, which, another revolution like that following the introduction of antiseptics, will be required to displace and render useless, as this and other modern works, which teach surgery from the standpoint of microbic pathology, have displaced and rendered useless the works of the days of "humoral pathology;" those days when "laudable pus" was regarded as a favorable prognostic. It almost makes one laugh now, to think of the assiduity with which suppuration was *encouraged;* to read of "healing by second intention;" of "mundifying and incarning," as taught by John Bell. But this is an age of progress—an age of electricity; and the surgery of to-day should be to that of last century as the electric light is to the sperm candles of our knee-breeched and shoe-buckled grand-grand sires of '76.

Mechanically, it is Appleton's *chef d'ouvre.*

---

WHAT TO DO IN CASES OF POISONING. By William Murrell, M. D. F. R. C. P. First American from Fifth English Edition. Edited by Frank Woodberry, M. D, Published by the MEDICAL REGISTER. Philadelphia, 1887. Price $1.00.

This is a most valuable contribution to literature, one which no practitioner should fail to keep on his desk, or rather in his satchel. It is practically arranged, so that in a moments time the desired information can be had, and at the same time, it is exhaustive, and comprises everything coming under the title of the book. The only defect which critics have yet pointed out, is the failure to mention transfusion of blood under certain circumstances. —B. E. H., M. D.

---

ANNUAL REPORT OF THE SPECIAL COMMITTEE ON SURGERY, TEXAS STATE MEDICAL ASSOCIATION, 1886, compiled and edited by Geo. Cupples, M. D., Chairman, Ex-President Texas State Medical Association, Ex-President West Texas Medical Association, Ex-Surgeon and Medical Director C. S. A., etc.

We have received a copy of this valuable work. It has been published in connection with, and is a part of, the Transactions of the Texas State Medical Association, at its last meeting, Austin, Texas, April, 1887. It is a royal quarto, printed in good style (Johnson Bros., San Antonio), on excellent paper, and contains, first, a list of the surgeons whose operations are therein tabulated; 2, a table showing all the amputations and the result; 3, disarticulations; 4, resections; 5, ligations of arteries, etc. Then, the removal of tumors, "noteworthy for site, size and character;" operations involving head and neck; involving thorax, abdomen—all classified; operations on rectum and anus; operations on male genital and urinary organs; ditto on female genital and urinary organs; operations involving bone and joints; involving organs of special sense; miscellaneous (unclassified) operations, and summary. The report makes 24 pages of tabulated matter, and two more pages are devoted to "Tables of the More Important Operations," including aggregate results of the reports of 1885 and 1886. From the latter we extract the following:

There were 626 major amputations, with 114 deaths, or 18.21 per cent; 185 minor ditto, with no deaths—total major and minor amputations, 811, of which 356 were "primary," 185 "secondary," and 122 "pathological;" the youngest patient (arm) being two years old, and the oldest (thigh) 78 years of age, with 114 deaths—a mortality of 14.56 per cent. Of the 356 primary operations, 22 were double amputations, 1 tripple, and 12 re-amputations. Thus, in the major amputations, 71 operations were performed on 35 patients, of which 26 patients recovered and 9 died—not a bad showing when we consider the circumstances under which operations are sometimes performed in the country.

Much credit is due Dr. Cupples for the manner in which this work has been done; it is a credit to Texas and to the State Medical Association.

---

A PRACTICAL TREATISE ON OBSTETRICS. Vol. IV. Obstetric Operations. The Pathology of the Puerperium. By A CHARPENTIER, M. D., Paris.Illustrated with lithographic plates and wood engravings. This is also Vol. IV of the "*Cyclopedia of Obstetrics and Gynecology*" (12 vols.), issued monthly during 1887. Price of the set $16.50. NEW YORK: WILLIAM WOOD & COMPANY.

We have already spoken of this work. When it is completed it will be an inexhaustible source from which all that is recent and reliable in obstetrics may be drawn. The handsome colored plates are highly commendable. The 12 Vols. are to be sold at the surprisingly low price of $16.50.

---

A CHART OF THE MUSCULAR SYSTEM, (according to Gray). Giving the origin, insertion, action and region of each muscle; also the nerve supplied to each; arranged by Dr. Drake. Published by R. Ferrand, Chicago. Copyrighted 1884. Price $3.

This is "anatomy in a nutshell." It is one of the most complete arrangements of the muscles, we have ever seen, and for ready reference, to refresh ones memory, say, on the eve of the performance of a surgical operation, is very useful. To the student of anatomy it is valuable; it enables him to get a general idea of the muscular system and its relation to the nerves, at a glance, while a close study of it will enable him to group the muscles in his mind, as they are there grouped;—thus making it easy to remember them. This chart is worthy of the attention of the profession, but an additional claim is that it is sold by, and for the benefit of, a worthy physician's *widow*.

---

A Practical Treatise on Renal Diseases and Urinary Analysis. By William Henry Porter, M. D., Professor of Clinical Medicine and Pathology in the New York Post-Graduate Medical School and Hospital ; Curator to the Presbyterian Hospital. One Vol. 360 pages, 100 illustrations. New York: William Wood & Company.

This is a valuable book. The faculty of this famous Institution hold appointments in most of the large Hospitals in New York, and their opportunities for observing and recording clinical facts in any disease, are extraordinary. Hence Dr. Porter may be regarded as excellent authority. We recommend the work to our readers.

---

Quiz Compend—Compend of Obstetrics: By Henry G. Landis, A. M. M. D. Late Professor of Obstetrics and Diseases of Women in Starling Medical College, etc. Especially adapted to the use of medical students and physicians; P. Blakeston Son & Co., Philadelphia, Pa., 1887. Cloth 116 pages. No price.

A Companion to the United States Pharmacopœa. Being a commentary on the last edition of the Pharmacopœa. With six hundred and fifty original illustrations. By Oscar Oldberg, Pharm. D., and Otto A. Wall, M. D., Ph. G. Second revised edition. New York: William Wood & Co., 1887.

This is a good book; it is a practical book. The first part is an alphabetical review of drugs. One can find at a glance what he wants to know about any given drugs without a world of speculation or theory; the latest accepted views are stated, in few words. It is well illustrated, also, especially that part which is devoted to the microscope in pharmacy. The "Weights and Measures" includes the old and the new systems, with rules for converting. The appendix is devoted to instructions how to prepare elixirs, etc. alltogether, it is a good book to have, and is well called "a companion to the U. S. P."

